# Early hCG addition to rFSH for ovarian stimulation in IVF provides better results and the cDNA copies of the hCG receptor may be an indicator of successful stimulation

**DOI:** 10.1186/1477-7827-7-110

**Published:** 2009-10-13

**Authors:** Peter Drakakis, Dimitris Loutradis, Apostolos Beloukas, Vana Sypsa, Vasiliki Anastasiadou, George Kalofolias, Helen Arabatzi, Erasmia Kiapekou, Konstantinos Stefanidis, Dimitris Paraskevis, Antonis Makrigiannakis, Angelos Hatzakis, Aris Antsaklis

**Affiliations:** 1 IVF Unit, Alexandra Hospital, 1st Department of Obstetrics and Gynecology, Athens University Medical School, Athens, Greece; 2 Department of Hygiene, Epidemiology and Medical Statistics, Medical School, University of Athens, Athens, Greece; 3 IVF Unit, Department of Obstetrics and Gynecology, Herakleion, Crete, Greece

## Abstract

A simple, safe and cost-effective treatment protocol in ovarian stimulation is of great importance in IVF practice, especially in the case of previous unsuccessful attempts. hCG has been used as a substitute of LH because of the degree of homology between the two hormones. The main aim of this prospective randomized study was to determine, for the first time, whether low dose hCG added to rFSH for ovarian stimulation could produce better results compared to the addition of rLH in women entering IVF-ET, especially in those women that had previous IVF failures. An additional aim was to find an indicator that would allow us to follow-up ovarian stimulation and, possibly, modify it in order to achieve a better IVF outcome; and that indicator may be the cDNA copies of the LH/hCG receptor. Group A patients (n = 58) were administered hCG and Group B rLH (n = 56) in addition to rFSH in the first days of ovarian stimulation. The number of follicles and oocytes and, most importantly, implantation and pregnancy rates were shown to be statistically significantly higher in the hCG group. This study has also determined, for the first time to our best knowledge, m-RNA for LH/hCG receptors in the lymphocytes of peripheral blood 40 h before ovum pick-up. cDNA levels of the hCG receptor after ovarian stimulation were significantly higher among women receiving hCG compared to those receiving LH. In addition, higher levels were encountered among women with pregnancy compared to those without, although this was not statistically significant due to the small number of pregnancies. It seems that hCG permits a highly effective and more stable occupancy of rLH/hCG receptors and gives more follicles and more oocytes. The determination of cDNA copies could be, in the future, a marker during ovulation induction protocols and of course a predictor for the outcome of ART in the special subgroup of patients with previous failures.

## Background

The achievement of a simple, safe and cost-effective treatment protocol in controlled ovarian hyperstimulation (COH) is of paramount importance to improve the quality of care in assisted reproduction. It is particularly important in the case of previous unsuccessful attempts. The midcycle gonadotrophin surge is a major event in the dynamics of ovulation. Rapidly increasing levels of luteinising hormone (LH) induce a number of key changes in both oocytes and follicular cells, which further modify the steroid and protein micro- and macroenvironment. These physiologic changes have a prominent role in the normal maturation of oocytes, the process of ovulation, and in subsequent fertilization and implantation [1].

Human chorionic gonadotrophin (hCG) has been used as a substitute for the LH surge because of the degree of homology between the two hormones [2]. hCG has a slower plasma metabolic clearance, which consists of a rapid phase in the first 5-9 h following intramuscular (IM) administration and a slower phase in the first 1-1.3 days after administration. Both LH and hCG are complex heterodimeric glycoproteins with a molecular weight of ~30 K for recombinant human LH (rLH) and 40 K for hCG. Their carbohydrate molecule is, though, different, thus leading possibly to a different affinity to the LH/hCG receptor and therefore to a differentiated function between LH and hCG. These two hormones have identical α-subunits and a high cysteine content. Most importantly, they have the same natural function--to cause ovulation and support lutein cells. The major differences between the two hormones include the sequence of the β-subunit, the regulation of the secretion of the two hormones, the carbohydrate component and the pharmacokinetics of clearance of hCG as opposed to LH [3,4].

The LH/hCG receptor has an almost ubiquitous distribution in reproductive organs, thus suggesting that the actions of hCG might be more extensive than previously thought. Independently of follicular stimulation hormone (FSH), low-dose hCG can support development and maturation of larger ovarian follicles that have acquired granulosa cell LH/hCG receptors, potentially providing effective and safer ovulation induction regimens. Human chorionic gonadotrophin seems to be capable of improving uterine receptivity by enhancing endometrial quality and stromal fibroblast function. Furthermore, through its actions on insulin-like growth factor binding protein-1 and vascular endothelial growth factor, hCG might stimulate endometrial angiogenesis and growth and extend the implantation window, thus increasing pregnancy rates [5,6].

Tailoring ovarian stimulation to the individual patient can be challenging because the ovarian response varies substantially between patients. Pharmacogenetics has emerged as a new area of research to improve the balance between desired and undesired actions of drugs, based upon the genetic predisposition of the individual patient.

The main aim of this study was to determine whether low dose hCG added to rFSH in regimens of ovarian stimulation could produce better results compared to the addition of rLH in women entering IVF-ET, especially in those women who had previous IVF failures. An additional aim was to find an indicator that would allow us to follow-up the ovarian stimulation and, possibly, predict a better IVF outcome in some women that may lead us to modify this stimulation; and that indicator may be the cDNA copies of the LH/hCG receptor.

## Methods

### Clinical study

This prospective, randomized, pilot study was designed to compare the IVF outcome between two groups of patients, the first receiving rLH and the second hCG, both in addition to rFSH in patients undergoing ovarian stimulation for IVF-ET. All patients had 2-6 previous failed attempts.

All patients attended our Unit within a period of 12 months. They all were between 36 and 42 years old, had a body mass index (BMI) of 32 or less, a menstrual cycle lasting between 21 and 35 days, normal serum levels of FSH, prolactin and TSH and a normal uterine cavity confirmed by hysteroscopy or hysterosalpingography. The causes for entering the program were: tubal factor, male factor, mild endometriosis (American Fertility Society classification stage I or II) [7] or unexplained infertility (with a history of at least 3 years of infertility). Patients hadn't had any other treatment with clomiphene citrate or gonadotrophins for at least 3 months before screening.

### Treatment protocol

Group A patients (final n = 58, two cycles were cancelled) were administered hCG in addition to rFSH in the first days of ovarian stimulation, while Group B patients (final n = 56, four cycles were cancelled) were administered rLH in addition to rFSH. Based on findings by Filicori et al. [8], the dose of hCG was chosen to be 200 IU IM in Group A given for four days. The initial dose of rLH was chosen to be 200 IU based on pharmacokinetic data for LH given for four days also.

Commercially available GnRH-a (Suprefact, buserelin; Hoechst, Frankfurt, Germany) was self-administered subcutaneously (sc) into the thigh at a dose of 200 μg/day, starting on the 2^nd ^day of the menstrual cycle and continuing until 24 h before the administration of hCG. Treatment with rFSH (Gonal-F; Serono, Geneva, Switzerland) was started on the third day of the menstrual cycle with 200 IU and continued until the administration of hCG for ovulation induction. rFSH was administered once daily as a sc injection in the abdomen. In Group A patients, 200 IU of hCG were also administered sc for the first five days of ovarian stimulation. In Group B patients, 200 IU of rLH were administered sc for the same number of days. The ovarian response was monitored by ultrasound and measurement of plasma E_2 _levels, while the dose of rFSH was adjusted accordingly [9]. The maximum dose allowed was 450 IU/day. The dose was reduced or discontinued if the patient was at risk of developing OHSS.

Ovulation was induced with 10 000 IU of hCG within 24 h after the last rFSH and GnRH-a administration, preferably when all of the following criteria had been met: the largest follicle had reached a mean diameter of at least 18 mm, at least one other follicle had a mean diameter of 16 mm, and serum E_2 _levels were within an acceptable range for the number of follicles present. Oocytes were retrieved by regular follicle aspiration 34-38 h after hCG injection. From one to three embryos were replaced in the uterine cavity on day 2 or 3 after OPU.

### Luteal phase support

Micronized progesterone (P_4_) (Utrogestan, Faran, Greece) (200 mg three times daily) was administered by the vaginal route as luteal phase support, starting after oocyte collection. P_4 _treatment was continued up to menstruation or for at least the first 3 weeks if the patient became pregnant. Definition of pregnancy required a positive βhCG test 14 days after embryo transfer. Definition of a clinical pregnancy required an endometrial gestational sac with a transvaginal ultrasound scan.

At the midluteal phase, careful abdominal ultrasound assessment was performed to record any signs of OHSS. The patient then was followed up, and the outcome (pregnancy or menstruation) was recorded.

### Method of assigning patients to study treatment

The randomization scheme was prepared by a computer using Proc PLAN in SAS version 6.12 (SAS Institute Inc., Cary NC).

### Safety evaluation

Safety was assessed through monitoring of all adverse events that occurred during the study, clinical assessment of local adverse reactions to injections, a questionnaire on clinical symptoms associated with OHSS at the time of hCG injection and at midluteal phase, u/s of the ovaries and abdomen and monitoring of any pathologic changes in routine laboratory values.

### Statistical methods

Patients were randomly assigned to rLH or hCG treatment according to balanced blocks of four subjects. Baseline characteristics of the patients were compared with the t-test or the Wilcoxon's rank sum test, as appropriate. Comparisons of pregnancy and other secondary outcomes between the two treatments were made by chi-square test or Wilcoxon's rank sum test, as appropriate. Multivariate logistic regression analysis was used to identify variables that are independent predictors of pregnancy outcome.

### IRB approval

The study protocol was approved by the Institutional Review Board or Ethics Committee of Alexandra hospital before screening the first patient. Written informed consent was asked before study entry, with the understanding that consent could be withdrawn by the patient at any time without prejudice.

### LH/hCG receptors

In this study we also examined the LH/hCG receptor mRNA expression in the peripheral blood. The LH/hCG receptor mRNA expression was determined in the lymphocytes of peripheral blood by a novel molecular beacon based real-time PCR assay (RT-PCR) 40 h before ovum pick up.

### RNA extraction

RNA extraction was performed as previously described [10]. Briefly, total RNA was extracted from the peripheral blood by employing a commercially available kit (RNA blood mini kit; Qiagen, Valencia, CA, USA) according to manufacturer's instructions. The use of RNase-free DNase I and carrier RNA, offered highly purified RNA.

### Reverse transcription-polymerase chain reaction (RT-PCR)

Total RNA extracted from peripheral blood was used for c-DNA synthesis by using Retroscript kit (Ambion, Austin, TX USA) according to manufacturer's instructions. Reverse transcription was followed by two rounds of nested PCR for LH/hCG mRNA and HPRT mRNA. Primer sequences used in both PCRs for LH/hCG mRNA amplification were designed with the Primer 3 program [11]. Primers pairs used for HPRT mRNA amplification have been described elsewhere [10]. All primers used were ordered from MWG Biotech (Table 1).

**Table 1:** PCR Primers

**mRNA**	**PCR primer** **pair**	**Primers** **5'-3'**	**Sequence**	**Annealing** **Temperature** **(°C)**	**Product** **size**
LH/hCG	Outer pair	Forward	CAA TGT GAA AGC ACA GTA AGG A	56	
		Reverse	AGG CTA TGA GCA GCA GAT AGA G	56	343
	Inner pair	Forward	GAA CTG AGT GGC TGG GAC TA	56	
		Reverse	GCA AAA GTC TGC AAA GGA GA	56	249
HPRT	Outer pair	Forward	CTCCGCCTCCTCCTCTGCT	50	
		Reverse	GCCTGACCAAGGAAAGCAAAG	50	528
		Forward	GCCGGCTCCGTTATGGCG	55	
	Inner pair	Reverse	AGCCCCCCTTGAGCACACAGA	55	226

The first round PCR mastermix contained 5 μl c-DNA in a total 50 μl volume. 5 μl of 10 × PCR buffer, 1.5 mM MgCl_2_/l, 0.2 μM of 3' and 5' outer primer, 0.2 mM of each dNTP/l and 1.5 u Taq polymerase were used (Invitrogen Life Technologies). Cycling conditions were 94°C denaturation, the temperature of annealing specific for primers and 72°C extension, with each step lasting 1 minute. Final extraction was performed at 72°C for 10 min. First PCR products were stored at -20°C.

For the second round of PCR 3 μl of the primary product were added to 47 μl freshly prepared mastermix containing PCR buffer, MgCl2, dNTPs, Taq polymerase and inner primers in the same quantities as the first one. The second round of PCR was performed for 30 cycles in the same cycling conditions with annealing temperature specific for inner primers. Products were stored at -20°C.

**Figure 1:**
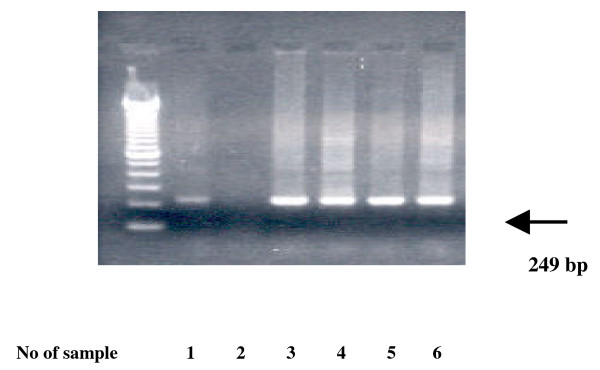
**Gel electrophoresis of RT-PCR products for hCG-R (fragment 249) in peripheral blood of women undergoing IVF-ET**. No 1 sample = positive control (cumulus cells), No 2 sample = negative control (distilled water), No 3-6 samples = peripheral blood of women undergoing IVF-ET.

The amplified products were analyzed by electrophoresis on 2% agarose gel containing ethidium bromide. 7 μl of each PCR product run in parallel with a 100 bp DNA ladder (Invitrogen Life Technologies). (Figure 1)

The presence of LH/hCG receptor mRNA was investigated by nested PCR in peripheral blood of women undergoing IVF-ET. A specific band of 249 bps corresponding to the LH/hCG receptor was detected in all women examined. Furthermore the presence of HPRT mRNA in peripheral blood confirms the integrity of the RT-PCR process.

### Real-time PCR

A real-time PCR assay was developed in order to quantitate LH/hCG receptor cDNA which was obtained with a reverse transcriptase (RT) assay from mRNA extracted from total blood.

Real-time PCR was optimized for human LH (hLH) receptor cDNA using a set of specific primers (inner set) and a molecular beacon probe labeled with a fluorescence dye for the amplification of hLH receptor. More specifically, hLH receptor primers and molecular beacon were newly designed targeting at a conserved region of gene sequence. The design of the primers and the molecular beacons was according to the standard requirements such as: (1) to avoid primer-beacon and primer-dimers and (2) the melting temperatures (T_m_) of the primers must be similar and at least 5-10°C lower than T_m_s of the molecular beacons. The software tool used for the assessment of the T_m _calculations was from the Virtual Genome Centre . The reaction mixture for RLT-PCR contained 2 μl LightCycler FastStart Taq Reaction Mix 10× (Roche, Molecular Biochemicals, Mannheim, Germany), 6 mM MgCl_2_, 0.75 μM primer hCG_for inner (forward) 5'-GAACTGAGTGGCTGGACTA-3', 0.75 μM primer hCG_rev inner (reverse) (Table 1), 5'-GCAAAAGTCTGCAAAGGAGA-3', 0.017 μM hLH_beacon 5'-**GCCGGC **CTGCTTACCCAAGACACCCCGATGTGCT **GCCGGC**-3' labeled with fluoresceine at the 5'-terminus and at their 3'-terminus bonded BHQ1, which is the quencher (Black Hole quencher 1). (MWG-BIOTECH Inc, U.K) (bold text indicates the complementary sequences forming the hairpin structure), 1.5 U FastStart Taq DNA polymerase (Roche, Molecular Biochemicals, Mannheim, Germany) and 1 U of uracil-DNA glycosylase (Roche, Molecular Biochemicals, Mannheim, Germany) in a final volume reaction 20 μl. Ten microliters of DNA sample DNA were added in the LightCycler 2.0 capillaries containing 10 μl of the reaction mix. The amplification conditions were optimized for LightCycler I as follows: one cycle of denaturation: 95°C for 10 min followed by 50 cycles of amplification at: 95°C for 10 s, 55°C for 10 s and at 72°C for 10 s. Uracil-DNA glycosylase was used to eliminate PCR 'carry over' contaminations from previous PCR reactions. The cycle number during which the fluorescence signal is above the background (CT) is proportional to the initial log concentration of the target DNA. For each run a standard curves was created in a 6-log range by 1:10 serial dilutions of hCG receptor's standard. The slope and correlation coefficient of each standard curve were calculated based on the average threshold cycle (C_T_) values measured in eight replicates for each dilution point ranging from 10^6 ^to 10^1 ^standard DNA templates. The PCR efficiency, E, corresponding to the experimentally derived dynamic range was computed as (10^-1/s ^- 1) 100, where s is the slope of the standard curve generated. The concentration of hCG receptor at unknown extracted DNA samples quantified using the standard curve for external standard ds and was expressed as copies per μl of cDNA.

### Preparation of standard DNA

hCG receptor amplicons were used as external standards after quantification in LightCycler 2.0 (Roche, Molecular Biochemicals, Mannheim, Germany) using PicoGreen dsDNA Quantification Kit (Molecular Probes). The concentration of hCG receptor amplicons was estimated according to standard curve calculated for serial dilutions of genomic DNA of known concentration (250-6.25 ng); Tenfold serial dilutions of the standards DNA with known DNA copy number were used for the generation of standard curves in amplification assays using molecular-beacon-based real-time PCR.

## Results

This randomized, pilot clinical study was performed in a study period of 12 months between January 2007 and December 2007. A total of 120 patients were enrolled and randomized. Six women did not complete the study; two presented at risk for OHSS and four failed to develop a follicle with a mean diameter of at least 17 mm. Of the remaining 114 women, 58 belonged to Group A (received hCG) and 56 to Group B (received rLH). All had at least one embryo transferred and all completed the midluteal phase assessment. The luteal phase was monitored in all patients who received hCG treatment.

The patients' characteristics prior to entering the program are summarized in Table 2. These characteristics did not differ between the two groups. The mean (SD) age of the patients was 36.4 (4.2) and 37.3 (1.8) for the hCG and LH group respectively (p = 0.147). BMI was similar in the two groups (mean (SD): 22.7 (3.0) and 23.7 (3.4) for hCG and LH respectively, p = 0.129). Mean (SD) serum FSH was 8.4 (3.1) IU/L for the hCG group and 8.2 (2.8) IU/L for the LH group, mean (SD) serum LH was 6.1 (2.1) and 6.4 (3.0) IU/L respectively and mean (SD) PRL was 12.2 (6.2) and 10.3 (4.7) ng/ml respectively for the two groups. The cause and duration of infertility, as well as the history of previous assisted reproductive techniques (ART) and non-ART pregnancies, were similar between the treatment groups. Uterine and ovarian sizes were comparable between the two groups (data not shown).

**Table 2:** Baseline characteristics of patients according to treatment group

	**hCG (N = 58)**	**LH (N = 56)**	**P**
Age (years), mean (SD)	36.4 (4.2)	37.3 (1.8)	0.147
BMI (kg/m^2^), mean (SD)	22.7 (3.0)	23.7 (3.4)	0.129
FSH (IU/L), mean (SD)	8.4 (3.1)	8.2 (2.8)	0.683
LH (IU/L), mean (SD)	6.1 (2.1)	6.4 (3.0)	0.623
PRL (ng/ml), mean (SD)	12.2 (6.2)	10.3 (4.7)	0.071
Years of Infertility (years), mean (SD)	6.7 (2.3)	6.9 (2.8)	0.677
Cause of infertility, %			
Tubal Factor	60	52	0.399
Male Factor	37	39	
Other	3	9	

The IVF outcome in the two groups is presented in Table 3. The mean (SD) number of days of ovarian stimulation was 10.8 (1.9) days for the hCG group and 11.1 (1.2) for the rLH group (p = 0.240). The mean (SD) total rFSH dose per patient was 2940 (1231) IU (hCG treatment) and 4261 (1090) IU (rLH group) (p < 0.001). Median (25^th^, 75^th^) serum E2 was 1888 (1119, 2118) and 720 (530, 1825) pg/ml for the hCG and LH group, respectively (p = 0.003).

**Table 3:** Impact of treatment on intermediate outcomes

	**hCG**	**LH**	**P**
Duration of stimulation (days) mean (SD)	10.8 (1.9)	11.1 (1.2)	0.240
E2 on the day of hCG (pg/ml) median (25^th ^- 75^th ^percentile)	1888 (1119, 2118)	720 (530, 1825)	0.003
Total rFSH dose/patient mean (SD)	2940 (1231)	4261 (1090)	<0.001
Number of follicles median (25^th ^- 75^th ^percentile)	7 (5,9)	4 (3,7)	<0.001
Oocytes number median (25^th ^- 75^th ^percentile)	6 (4,7)	3 (2,6)	<0.001
Proportion of mature oocytes (%) median (25^th ^- 75^th ^percentile)	75.0 (57.1,100)	66.7 (66.7,100)	0.752
Proportion of fertilized oocytes (%) median (25^th ^- 75^th ^percentile)	71.4 (66.7, 80.0)	66.7 (50.0,100)	0.317
Number of transferable embryos median (25^th ^- 75^th ^percentile)	4 (2,4)	2 (1,3)	<0.001
Proportion of patients with endometrium thickness >8 mm (%)	80	69	0.190
Implantation rate (%)	8.9	4.4	0.125

### Oocyte retrieval, fertilization and embryo cleavage

The median number of follicles was 7.0 for hCG and 4 for LH (p < 0.001) and the median number of oocytes retrieved per group was 6 for the hCG group and 3 for the LH one (p < 0.001). No patient had more than 14 oocytes.

The majority of oocytes were in metaphase II (75.0% for the hCG group and 66.7% for the rLH group, p = 0.752. The percentage of nuclear maturity was thus comparable between rLH and hCG

There was no significant difference in fertilization rate between the two treatment groups (71.4% for hCG vs. 66.7% for LH, p = 0.317).

The median number of transferable embryos was 4 for the hCG group and 2 for the rLH group (p < 0.001).

The mean number of embryos transferred was 2.4 ± 0.4 for the hCG group and 2.5 ± 0.4 for the LH group (p = 0.185) (Table 3).

Only embryos transferred during the study treatment cycle were considered in this analysis. All 114 patients had at least one embryo transferred. No more than three embryos were replaced in any patient.

### Endometrium

The percentage of patients with endometrial thickness > 8 mm was slightly higher in the hCG group (80% compared to 69%; p = 0.190).

### Implantation rate

Implantation rate (i.e. total pregnancies over the total number of transferred embryos) was 8.9% for the hCG group and 4.4% for the rLH group p = 0.125 (Table 3). No monozygotic were found in this study.

### Pregnancy rate

In a univariate analysis, we assessed the effect of parameters such as age, BMI, basal serum hormone values and treatment with either hCG or LH on the achievement of pregnancy (Table 4). Treatment with hCG was found to be the only parameter that significantly increases pregnancy outcome expectation. A total of 16 clinical pregnancies (27.6%) were recorded for hCG patients while 6 pregnancies (10.7%) were recorded for the rLH group (p = 0.022) (Table 4).

**Table 4:** Univariate associations of patients' characteristics and treatment with pregnancy outcome

	**Pregnancy**		
	**Yes**	**No**	**P**
Age, mean (SD)	36.6 (3.3)	36.9 (3.3)	0.736
BMI, mean (SD)	23.1 (3.8)	23.2 (3.1)	0.934
FSH, mean (SD)	7.28 (2.98)	8.55 (2.93)	0.071
PRL (SD)	11.9 (5.2)	11.1 (5.6)	0.551
LH (SD)	6.29 (2.53)	6.24 (2.60)	0.944
Treatment, n (%)			
hCG	16 (27.6)	42(72.4)	0.022
LH	6 (10.7)	50 (89.3)	

We also observe that (Table 5), after having adjusted for potential differences in age, BMI and baseline PRL, FSH, E2, women receiving hCG treatment have 3.6 times higher probability of achieving pregnancy compared with women receiving LH (95% CI: 1.21-10.71, p = 0.022).

**Table 5:** Multiple logistic regression model for potential factors associated with increased probability of pregnancy

	**Odds ratio**	**95% CI**	**P**
Age (/year)	1.03	(0.00, 1.20)	0.735
BMI	1.02	(0.87, 1.19)	0.849
Therapy hCG/LH	3.60	(1.21, 10.71)	0.022
PRL	1.01	(0.92, 1.11)	0.865
FSH	0.84	(0.70, 1.02)	0.078
E2	1.00	(1.00, 1.00)	0.746

### Ovarian hyperstimulation syndrome and adverse events

The proportion of patients presenting with moderate OHSS [12] was similar in the two groups (about 12%) and no serious adverse events were noted in any group.

### cDNA copies of the hCG receptor

cDNA levels after ovarian stimulation were significantly higher among women receiving hCG compared to women receiving LH (median levels: 25.3 copier per μl of cDNA vs 6.8 copier per μl of cDNA, respectively, p = 0.012) (Table 6).

**Table 6:** cDNA levels according to treatment and pregnancy outcome

	**cDNA (copies/μl)** **median (25^th ^- 75^th ^percentile)**	**P**
Treatment, n(%)		
hCG	25.3 (4.5,126.0)	0.012
LH	6.8 (1.4, 48.5)	
Pregnancy, n(%)		
Yes	14.2 (4.5, 72.9)	0.687
No	9.0 (2.6, 92.9)	

As far as prediction of pregnancy outcome by cDNA levels alone, higher levels are encountered among women with pregnancy compared to those without, this is not statistically significant though (Table 6).

## Discussion

The necessary and the optimal dose as well as the time of LH administration in IVF cycles for the achievement of good quality oocytes and embryos has not yet been determined. The two-cell theory suggests that both FSH and LH are needed for normal follicular growth and maturation, but until now the main role has been attributed to FSH.

Studies in non-human primates have revealed that LH may act by increasing intraovarian androgens which promote FSH responsive granulosa cell function [8,13] and, previous studies in humans have shown that LH acts synergistically with FSH to promote follicular growth [14]. However, no data exist on the potential clinical benefit of an "LH priming" effect as well as hCG.

Several studies have demonstrated that the administration of LH activity combined with FSH can exert significant actions on folliculogenesis. Still, reports on clinical data have been controversial. Late reports have employed rLH either before the administration of rFSH [6] or during the late part of ovarian stimulation [8,15,16]. A beneficial induction effect has been shown when rLH is administered before rFSH commenced in IVF cycles [6] but the authors suggested that the effect of supplementation with rLH on the clinical outcome needs to be clarified in the future. Our previous data with human luteinising hormone supplementation in the beginning of ovarian stimulation showed a beneficiary effect [17] and this effect is with the early addition of hCG in this study.

The action of LH in preantral and small antral follicles was reported in literature to be limited [18]. However, granulosa cells express LH/hCG receptors and can be stimulated by both FSH and LH [19]. It has also been demonstrated by us that mRNA for the FSH and LH receptors exists in denuded oocytes as well as in preimplantation embryos at different stages, indicating a physiological role of LH in the oocyte maturation process and early embryonic development in the mouse and in humans [10,11]. Indeed, luteinising hormone activity can be provided in various ways: by human-derived LH contained in hMG, by recombinant LH, and by human-derived or recombinant hCG.

Our study has shown, for the first time to our best knowledge, that the administration of 200 IU of hCG daily, in addition to rFSH, is a safe and possibly better alternative of human recombinant luteinising hormone supplementation for patients undergoing IVF/ICSI-ET. As stated before, other investigators have already used 200 IU of hCG in the last 3-4 days of ovarian stimulation in the long protocol [8,15,16]. In our study we used hCG supplementation during the first five days of ovarian stimulation in the short protocol. No negative impact of low-dose hCG administration was detected in patients receiving this treatment.

We also have to note that if we analyse the results with intention to treat, that is including the cases of patients that dropped treatment, we still have a statistically significant difference between pregnancy rates (p = 0.018).

In most of the currently used ovarian stimulation protocols, serum LH is clearly suppressed through pituitary down regulation by GnRH agonists or antagonists. In the short protocol, LH is suppressed during the final days of the follicular growth (days 7-11). It seems, from our study, that the administration of 200 IU of hCG daily from days 3- 7 during the follicular growth permits a sufficient LH level in this period of ovulation induction. Our results have shown that with hCG we had lower number of gonadotrophin ampoules used, higher fertilization rate, higher and a better pregnancy rate with a tendency for a better implantation rate. In addition, the percentage of mature oocytes and the number and quality of embryos was comparable between rLH and hCG, thus showing that hCG, in the specific dose and way of administration, had no harmful effect on ovarian stimulation.

An explanation of the better ovulation profile in hCG treatment cycles could be the different isoform of hCG as compared to rLH. Differences in the carbohydrate moiety may make the molecule more sensitive to the binding receptor. In addition, the longer plasma half-life of hCG (half life of hCG is 33 hours while half life of rLH is 10-12 hours) results to a better and prolonged effect in the ovarian stimulation process [3,20]. It seems, therefore, that its longer plasma half-life and its greater potency (roughly six to eight times greater than that of LH) permit highly effective and more stable occupancy of the LH\hCG receptors. The fact that serum E_2 _levels in patients who received rLH were statistically significantly lower than in patients treated with hCG, shows that indeed the occupation of the LH/hCG receptor in the rLH-administered patients is less compared to the hCG stimulated patients.

The fact that single doses of rLH have a shorter effect than those of hCG is further substantiated by the performance of rLH in relation to the main characteristics of a natural LH surge as reported in the literature. The natural surge lasts for about 2 days (49 ± 9 h) and is composed of an ascending phase (around 14 h), a plateau (around 14 h), and a descending phase (around 20 h) [21]. LH serum levels, when measured by RIA, are about 10-20 times the basal LH levels. The surge profile obtained after a single injection of 5,000 IU hCG is very different from that of the natural LH surge. The main differences are the total duration (the hCG surge can last up to 120 h) and the length of the descending phase. Previous publication suggested that using a conversion factor of 2.5, a dose of 12,500 IU rLH would be as effective as 5,000 IU hCG in humans [22].

In terms of safety, this study shows that 200 IU of hCG is a well tolerated dose. Reported adverse effects are similar to those usually reported during stimulation cycles with rFSH and hMG. No negative impact of low-dose hCG administration was detected in patients receiving this treatment. Our study, thus, demonstrated that the administration of 200 IU of hCG daily can be applied as a supplement in patients with previous failed attempts during the short protocol in the first 5 days of superovulation.

This study has also determined, for the first time to our best knowledge, m-RNA for LH/hCG receptors in the lymphocytes of peripheral blood 40 h before ovum pick up. cDNA levels of the hCG receptor after ovarian stimulation were significantly higher among women receiving hCG compared to women receiving LH. In addition, higher levels were encountered among women with pregnancy compared to those without, although this was not statistically significant due to the small number of pregnancies. It seems that hCG permits a highly effective and more stable occupancy of rLH/hCG receptors and gives more follicles and more oocytes. This is due to the "down regulation" mechanism that is caused by the effect of the hormone on the receptors. As far as hCG and rLH are concerned for ovarian simulation, from this pilot study, it seems that the addition of hCG in the short protocol helps to improve the ovulation profile. It remains to be clarified, with the successive measurements of hormone in the blood during ovulation induction, which range of hCG in the blood relates with the optimum number of receptors.

The LH/hCG receptor has an almost ubiquitous distribution in reproductive organs, thus suggesting that the actions of hCG might be more extensive than previously considered [22,23]. The localization of the LH/hCG receptors in extragonadal reproductive tissues has suggested that hCG might exert additional actions and that these mechanisms could be exploited to enhance the efficacy of treatment used to manage infertile patients. The expression of LH/hCG receptors by theca cells and by granulosa cells has been well characterized [23,24]. Low-dose hCG can also be used instead of LH to permit in a more sustained manner the progression of folliculogenesis [5]. Moreover, the tendency, although not statistically significant for this sample number, for better implantation rate noted with hCG may reflect a more beneficial action to the endometrium compared to that of rLH administration since the number of embryos and their quality was the same.

However, the importance of this factor for the moment is not clearly established. The cDNA copies of LH/hCG receptor may act in concert with other environmental and genetic factors that could contribute to improve the ovulation protocols in the future. Also the determination of cDNA copies could be, in the future, a marker during ovulation induction protocols and of course a predictor for the outcome of ART in special subgroup of patients with previous failures. We are currently determining the variation of cDNA copies from the start of ovarian stimulation till luteal phase to see if there is a correlation between their level throughout ovarian stimulation and pregnancy outcome and if stimulation can be adjusted accordingly in order to provide a better outcome.

Finally, the results in this study are in agreement with the conclusion published recently by Filicori et al [5] stating that greater understanding of the physiologic role that hCG might play in human reproduction is beginning to suggest novel therapeutic applications for this traditional hormone of pregnancy.

## Conclusion

Our study, as stated previously, demonstrated that the administration of 200 IU of hCG daily can be applied in addition to rFSH in patients with previous failed attempts during the short protocol in the first 5 days of superovulation increasing, thus, pregnancy rate.

This study has also determined, for the first time to our best knowledge, m-RNA for LH/hCG receptors in the lymphocytes of peripheral blood 40 h before ovum pick up. cDNA levels of the hCG receptor after ovarian stimulation were significantly higher among women receiving hCG compared to women receiving LH. In addition, higher levels were encountered among women with pregnancy compared to those without, although this was not statistically significant due to the small number of pregnancies. The determination of cDNA copies could be, in the future, a marker during ovulation induction protocols and of course a predictor for the outcome of ART in special subgroup of patients with previous failures.

## Competing interests

The authors declare that they have no competing interests.

## Authors' contributions

PD, DL, AM and AH made substantial contributions to conception and design and also to interpretation of data. AB, VS, VA, KG, HA, EK, KS and DP made substantial contributions to acquisition and analysis of data. PD was involved in drafting the manuscript. AA gave final approval of the version to be published. All authors read and approved the final manuscript.
